# Short-Term Exposure Effect of Ambient Fine Particulate Matter, Ozone and Cold Temperature on Emergency Room Visits for Asthma Patients

**DOI:** 10.3390/toxics11020094

**Published:** 2023-01-19

**Authors:** Chun-Gu Cheng, Shang-Yih Yen, Chih-Chun Hsiao, Yen-Yue Lin, Yin-Han Chang, Yu-Hsuan Chen, Chun-An Cheng

**Affiliations:** 1 Department of Emergency Medicine, Taoyuan Armed Forces General Hospital, Taoyuan 32549, Taiwan; 2 Department of Emergency Medicine, Tri-Service General Hospital, National Defense Medical Center, Taipei 11490, Taiwan; 3 Department of Neurology, Tri-Service General Hospital, National Defense Medical Center, Taipei 11490, Taiwan; 4 Department of Nursing, Taoyuan Armed Forces General Hospital, Taoyuan 32549, Taiwan; 5 Department of Psychology, National Taiwan University, Taipei 10621, Taiwan; 6 Division of Chest Medicine, Department of Internal Medicine, Cheng Hsin General Hospital, Taipei 11220, Taiwan

**Keywords:** air pollutants, cold temperature, short-term exposure, asthma emergency room visits

## Abstract

(1) Background: The acute effects of ozone, cold temperature and particulate matter less than 2.5 μm (PM_2.5_) in size related to asthma attacks are well known worldwide. The adverse effects of ozone and cold temperature on asthma morbidity in Taiwan are still inconclusive. (2) Methods: This retrospective study included patients who had asthma emergency room visits (ERVs) from 1 January 2016 to 31 December 2019 in a regional hospital in Taiwan. The short-term negative effects were estimated using Distributed Lag Non-Linear Models (DLNMs) for the relative risks (RRs) of asthma ERVs associated with PM_2.5_, ozone and cold temperature exposures within 5 days. (3) Results: There was a significant association between a 10 ppm increase in PM_2.5_ exposure and asthma ERVs at a 2-day lag (RR 1.166, 95% confidence interval (C.I.): 1.051–1.294). There was a significant association between ozone and asthma ERVs at a 1-day lag (RR 1.179, 95% C.I.: 1.034–1.345). The ambient temperature in cold weather compared with the temperature of minimum asthma ERV showed an RR of 1.214, 95% C.I.: 1.009–1.252 at a 1-day lag. (4) Conclusions: This study provides evidence that short-term exposure to fine suspended particulates, ozone and inverse temperature is associated with asthma exacerbation.

## 1. Introduction

Asthma is a chronic respiratory disease that affects more than 350 million people worldwide, and its incidence is on the rise. It is reported to kill approximately 383,000 people each year [1]. Acute asthma attacks cause dyspnea, which requires emergency management visits to emergency departments, resulting in a healthcare burden. Acute asthma exacerbation is an important public health problem that induces patients’ respiratory discomfort and increases burdens on their families, health professionals, health care institutions and even the development of the nation [2]. Asthma contributed 13.2 million years of life lived with disability and 10.5 million years of life lost due to premature death across all ages, as reported by the World Health Organization (WHO), in 2016 [3].

For chronic asthma, the estimated prevalence is the highest in the African region (11.3%), but the lowest in the Southeast Asia region (8.8%) [3]. Up to 36% of children younger than 18 years old with asthma reported emergency department visits for asthma in the past year in the United States [4]. The prevalence of asthma was 11.53% in Taiwan in 2011 [5]; the estimated number of asthma patients was approximately 2 million. The prevalence rate of asthma in children under 12 years old was 5.6%, and 9.2% of them went to the emergency department due to an asthma attack within one year, according to the National Health Interview Survey as reported by the Taiwanese Ministry of Health and Welfare National Health Service [6]. The 55.1/100,000 hospitalization rate of asthma is still higher than other countries, and policies are needed to improve the result [7].

Exposure to air pollution can lead to respiratory and cardiovascular diseases, lung cancer [8,9,10,11] and sudden hearing loss [12]. Particulate matter (PM) is the largest cause of air pollution; the higher the concentration of fine suspended particulates (PM_2.5_) in the air, the higher the relative risk of respiratory diseases [13]. In 2013, the International Agency for Cancer (IARC) listed PM_2.5_ as a first-class carcinogen [8].

Motor vehicle combustion, fossil fuel (traffic exhaust), power plants or other industrial processes may be the main sources of PM_2.5_ and ozone (O_3_). Traffic and power generation are the main sources of urban air pollution, and traffic-related air pollution (TRAP), as well as the air pollutants O_3_, nitrogen dioxide (NO2) and PM_2.5_, can induce airway inflammation [14].

A past study found that PM_2.5_ was related to asthma emergency room visits (ERVs) in Taipei [15]. Younger patients, below 18 years old, with asthma had significant risk associations with ambient levels of PM_2.5_ and ozone in southern Taiwan [16]. Studies on the associations between ambient temperature and ERVs vary due to differential areas with different climates and terrains [16,17,18,19]. Females and younger patients were more vulnerable to the effects of low temperatures. Adults are at a higher risk of asthma ERVs associated with extremely high temperatures due to outdoor work [16].

Taoyuan City is an urban area of Taiwan located in southern Taipei, and New Taipei cities have higher TRAPs. Longtan District is located in southern Taoyuan. In the special terrain surrounding the U sharp mountain, the seasonal wind carries air pollutants from mainland China and Linkou District that is difficult to clean out in the winter. Several factories for wafer manufacturing which use ozone water for wafer washing are located in the Longtan District. The air pollution and climate factors that may cause asthma attacks with delayed effects need to be studied. The relationship between asthma ERVs and air pollution or climate conditions in special areas is worthy of study.

The aim of this study focused on the relationship between asthma ERVs, ambient pollutants and climate conditions for a period of four years in Taiwan. The unique terrain caused air pollutants to gather via seasonal winds in winter, which resulted in asthma attacks. Although the government has focused on reducing air pollution for years, some air pollutants are still not targeted by the WHO’s air pollution guideline values, and aggressive policies to reduce them that would promote citizens’ health have not yet been introduced.

## 2. Materials and Methods

We analyzed daily asthma ERVs data for the time period 1 January to 31 December from 2016 to 2019 at Taoyuan Armed Forces General Hospital, Taoyuan, Taiwan. The regional hospital is located within the southern area of Taoyuan City in Taiwan and has more than 60,000 ERVs yearly; it is the only hospital with an emergency department in southern Taoyuan City. The Longtan District is a sector of hilly terrain located in the southern region of Taoyuan, with the highest height in the southeastern part, the lowest height in the northwestern part, higher terrain on both sides and 124,442 residents in 2021 [20].

The daily air pollutant gas data were obtained from the Taiwanese Environmental Protection local air quality monitoring station in Longtan of Taoyuan City (24°51′50.8″ N, 121°13′00.2″ E), which is the nearest station (approximately 2.35 km) to Taoyuan Armed Forces General Hospital. Air pollutants include PM_2.5_, O_3_, NO_2_, sulfur dioxide (SO_2_), carbon monoxide (CO), relative humidity (RH) and ambient temperature. The data were obtained from the Environmental Protection Administration’s (EPA) Taiwan Quality Monitoring Network [21].

**Figure 1:**
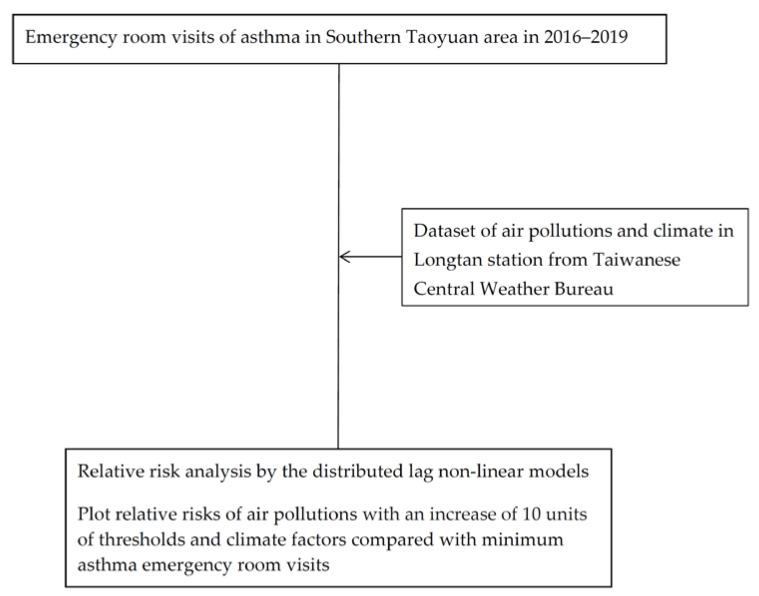
The flowchart in this study.

We used ERVs data, including information from 1 January 2016 to 31 December 2019. The occurrence of ERVs was retrieved via International Classification of Diseases, 9th Revision, Clinical Modification (ICD-9-CM) codes 493 and ICD-10-CM code J45. The flowchart of this study is described in Figure 1. This study was approved in TSGH by the TSGHIRB No.: C202105149.

Descriptive statistics of asthma ERVs, air pollutants and meteorological factors were evaluated (Table 1). The distributed-lag nonlinear model (DLNM) was performed for the association between asthma ERVs, air pollutants and meteorological factors (PM_2.5_, PM_10_, O_3_, CO, NO_2_, SO_2_, air temperature and relative humidity). The correlations between air pollutants and meteorological factors were assessed. We set the exclusion criteria at |r| > 0.8 to reduce the collinearity problem between each variable. The correlations were above 0.8 between PM2.5 and PM10 as well as between NO_2_ and CO, and we excluded PM10 and CO (Table 2). The levels of SO_2_ were not more than 10 units higher than the median and were excluded. The relation of ERVs and individual air pollution (PM_2.5_, O_3_, NO_2_) and air temperature used multivariable DLNM analysis-adjusted wind speed, relative humidity, holiday, seasonality and time trends, and lag was set at 0–5 days [22]. The DLNM handled the time series data for nonlinear relationships and delayed effects. The Poisson regression managed the count data using a generalized linear model. The relative risk (RR) for asthma ERVs, related to the levels of ambient pollutants and the air temperature, were analyzed by a quasi-Poisson regression model with a linear function evaluating the exposure–response function. The maximum lag was set to 5 days for each variable. The natural cubic spline used for air temperature, relative humidity and wind speed had 4 equally spaced internal knots. We adjusted the seasonality and time trends using a natural cubic spline with seven degrees of freedom (df) for the calendar time. The factor variables of days of the week and holidays were adjusted. Thresholds were arranged as the median of air pollutants and minimum asthma ERV temperature (MAT) (26 °C). RRs of air pollutants were assessed as a 10-unit increase from the reference levels. The cold air temperature was 25% of the air temperature in research years (18.1 °C), and the RR of air temperature used ERVs at 18 °C compared with ERVs at 26 °C. The extremely cold air temperature was 1% of the air temperature in the research years (9.8 °C), and the RR of air temperature used ERVs at 10 °C compared with ERVs at the reference level of air temperature. The analysis was performed by the R package dlnm (R version 4.0.2) [22]. Poisson distribution-like schematic calculation of possible variables for the relative risk of ERVs was conducted. Poisson regression is a generalized linear model that assumes that the response variable Y and the logarithm of its expected value can be modeled by a linear combination of air pollutant parameters. The average asthma ERVs, air pollutants and meteorological factors each month are shown in a graph.

We counted the numbers of asthma ERVs in pediatric (0–17 years old), adult (18–64 years old) and older asthma patients (>65 years old). According to sex, we also counted the numbers of asthma ERVs in male and female patients. The cold season was defined as November to April, and the warm season was defined as May to October.

## 3. Results

**Figure 2:**
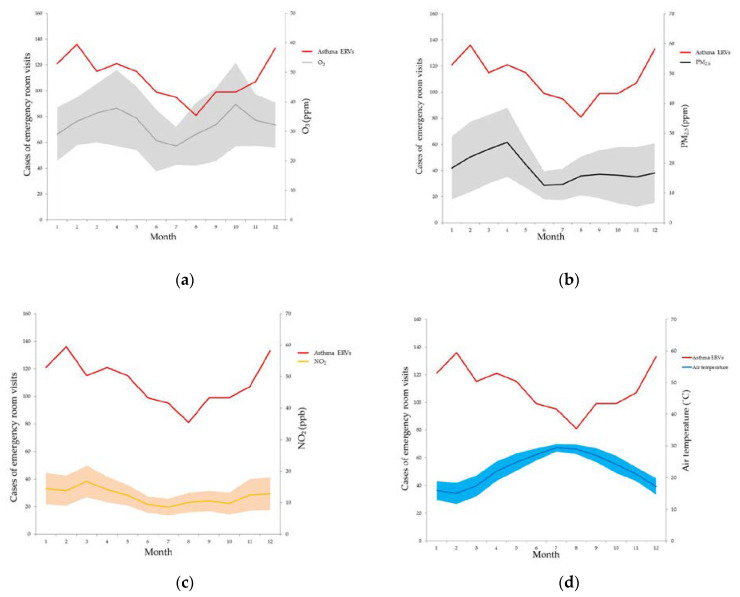
The asthma emergency room visits, ozone (**a**), PM_2.5_ (**b**), NO_2_ (**c**) and air temperature (**d**) in different months over four years in the Longtan district of Taoyuan in Taiwan.

There were 1321 asthma ERVs in this study. The average age at the time of the asthma ERVs was 43.43 ± 29.44 years old. There were 679 males and 642 females in the four-year period. A total of 999 (75.62%) asthma ERVs were discharged after treatment, 296 (22.41%) asthma ERVs required hospitalization, 9 asthma ERVs were transferred to other hospitals, 19 asthma ERVs were discharged against medical advice and 1 asthma ERV resulted in death (Table 1). The maximum number of monthly asthma ERVs was 136 in February, and the minimum was 82 asthma ERVs in August. The (lowest) nadir mean air temperature was 15.03 °C in February and the peak was 29.37 °C in July. The peak of the mean PM_2.5_ was 26.97 ppm in April. The mean O_3_ values exhibited twin peaks of 37.89 ppb in April and 39.09 ppb in October (Figure 2). 

**Figure 3:**
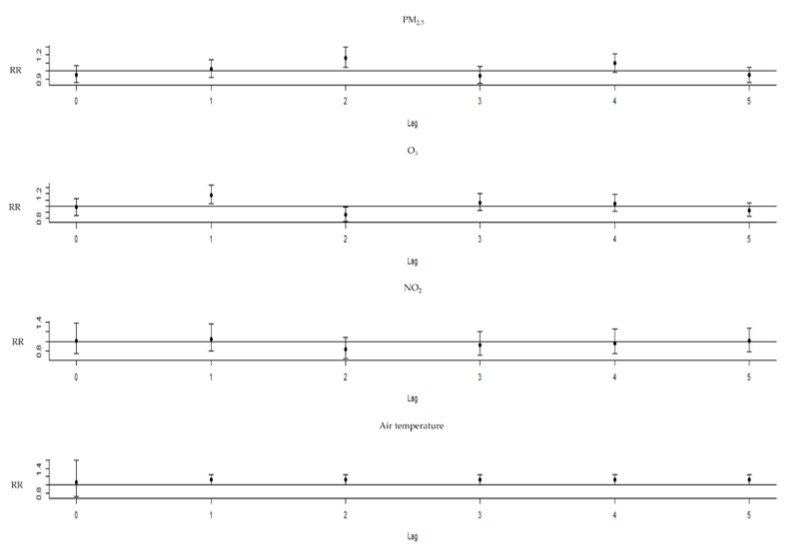
The relative risks of PM_2.5_, ozone and NO_2_ with the thresholds increased every 10 units, and air temperature compared with the temperature of minimum asthma emergency room visits.

**Figure 4:**
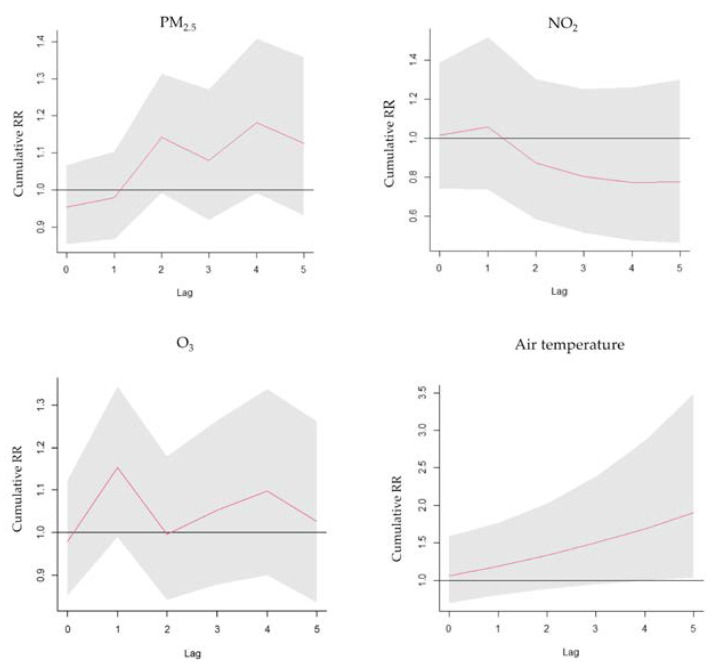
Lag−response curve of the cumulative effects of PM_2.5_, O_3_, NO_2_ and air temperature.

PM_2.5_ exposure had a positive relationship with asthma ERVs, with an RR of 1.166 (95% confidence interval (C.I.): 1.051–1.294) for a 10-unit increase with a 2-day lag. O_3_ exposure had a positive relationship with asthma ERVs, with an RR of 1.179 (95% C.I.: 1.034–1.345) for a 10-unit increase with a 1-day lag. The ambient temperature in cold weather (25%:18 °C) was compared with MAT of 26 °C (RR 1.214, 95% C.I.: 1.009–1.252) with a 1-day lag. NO_2_ exposure had no significant relationship with asthma ERVs, with an RR of 1.238 (95% C.I.: 0.881–1.74), for a 10-unit increase with a 1-day lag (Figure 3). The lag−response curve of incremental cumulative effects of air temperature showed a cumulative RR of 1.9 (95% C.I.: 1.035–3.488); a cumulative effect of PM_2.5_ of 1.125 (95% C.I.: 0.933–1.358); O_3_ of 1.027 (95% C.I.: 0.836–1.262) and NO_2_ of 0.776 (95% C.I.: 0.464–1.293) (Figure 4). The RR of PM_2.5_ in the differential age group was 1.195 (1.001–1.426) at a 2-day lag in pediatric asthma patients, 1.253 (95% C.I.: 1.037–1.515) at a 1-day lag in older asthma patients and 1.281 (95% C.I.: 1.087–1.509) at a 4-day lag in adult asthma patients. The extremely cold temperature (1%: 10 °C)) association was evident in elderly asthma patients, with an RR of 1.378 (95% C.I.: 1.049–1.811) compared with MAT at a 1-day lag and an RR of 1.193 (95% C.I.: 1.037–1.373) at a 1-day lag in male asthma patients, whereas no association was observed among children or females.

The RR of PM_2.5_ was 1.231 (95% C.I.: 1.061–1.244) at a 2-day lag, and the RR of O_3_ was 1.33 (95% C.I.: 1.106–1.599) at a 1-day lag every 10-unit increase in female asthma patients. The RR of NO_2_ in the warm seasons was 2.308 (95% C.I.: 1.132–4.705) every 10 units, without lag. The RR of PM_2.5_ in the cold season was 1.2 (95% C.I.: 1.051–1.37) every 10 units, with increased lag at 2 days; the RR of O3 in cold seasons was 1.222 (95% C.I.: 1.007–1.484) every 10 units, with increased lag at 1 day and the RR of cold temperature (13 °C) compared with 18 °C in cold seasons was 1.129 (95% C.I.: 1.027–1.24) at a one-day lag.

## 4. Discussion

Our study suggested that the ozone and cold weather related to asthma ERVs are associated with PM_2.5_. Chemicals with oxidant-generating capabilities affect the human respiratory and immune systems [23]. Recent studies in cells and animal models have suggested several possible mechanisms, of which the most consistent observation is the direct effect of particulate components on the production of reactive oxygen species (ROS) and the resulting oxidative stress and inflammatory responses [24,25].

Asthma exacerbation is defined as a worsening of shortness of breath, cough, wheezing, or chest tightness. If patients don’t receive effective treatment immediately, it will increase airflow resistance, causing an increase in breathing workload, gas exchange inefficiency and respiratory muscle fatigue and result in hypercapnic and hypoxemic respiratory failure. Acute asthma attacks are a significant public health problem and influence quality of life, affecting patients and families through causing absences from labor and school, frequent ERVs, hospitalizations and possible death [2,26].

PM carries metal components, and organic matter has the ability to generate oxygen free radicals, which can stimulate cells to produce ROS [27]. ROS-induced oxidative damage to lung cells may be the primary cause of damage due to PM exposure. PM_2.5_ may contain toxic substances from combustion, including acids, metals and nitrates. These ingredients can accumulate in the lungs and cause allergies with increased immunoglobin E and inflammation [28,29]. PM_2.5_ concentrations have been shown to be higher in cold weather [30], and our study also found the same trends. A previous study found that PM_2.5_ was related to asthma ERVs in Taipei [15]. Our study showed similar findings. The Longtan District had a higher altitude, from 150 m in the northwestern part to 230 m above sea level in the southeastern part, and is surrounded by mountains on the eastern, southern and western borders. The seasonal wind from the northeast carries the pollutants from mainland China, and the LinKou thermal power plant causes higher PM_2.5_ in the winter season. The mean daily PM_2.5_ concentration is at its highest in April due to residents worshiping their ancestors by burning incense and joss paper during the Qingming Festival on 5 April. The highway crosses the Longtan District with higher TRAPs. A lag of only one day in PM_2.5_ exposure was associated with acute asthma attacks in older asthma patients, which means that older asthma patients are more sensitive to PM_2.5_ changes and have weaker respiratory tract defenses than pediatric and adult asthma patients. Adult asthma patients carrying a heavy workload could initially go to the clinic and be sent to emergency departments, but symptoms would worsen after several days, and this would cause an even longer lag for asthma ERVs.

Ozone, a gas, is one of the most common air pollutants. It is most common in cities where there is heavy road traffic [14]. It is also more common in the spring and fall, when there is more sunlight due to higher attitude and low wind photochemical reaction from NO_2_. Ozone triggers asthma because it is very irritating to the lungs and airways. It is well-known that ozone concentration is directly related to asthma attacks, reducing lung function and causing ERVs to be required for medication treatment [31]. Acute ozone exposure in men causes sputum neutropenia in 30% of subjects, especially young children, women and those with persistent cardiorespiratory disease [32]. Exposure to O_3_ can promote airway infections and increase the risk of asthma attacks [33,34]. A similar finding was noted in our study. Inhaled ozone does not enter cells, but reacts with components of the airway-lining fluid in order to generate other ROSs and enhance local oxidative stress, inflammation and epithelial cell injury [35]. The mean concentration of ozone was five units higher than that in Taipei [12]. The potential reason was that several wafer manufacturing factories using ozone water for wafer washing were located in Longtan district. A past study found that daily O_3_ concentrations were associated with a higher risk of asthma in young adults. Women aged 40 to 64 years had the highest relative risk of ERVs, with an RR of 1.21 (95% CI 1.05–1.39) [16]. However, our study showed different findings, because as adult asthma patients need to work, they tend to go to the clinic rather than the emergency department for treatment. O_3_ was related to asthma ERVs in the female patients and in the cold seasons in our study.

Extreme temperatures are related to ERVs in the United States [36], and cold temperature-related respiratory ERVs in Taiwan are similar [37]. The link between extreme temperatures and asthma risk only occurs during ERVs and outpatient visits [16]. Cold temperatures in crowded environments may exacerbate transmission and lead to cross-infection [19]. Exposure to extreme temperatures may increase pulmonary vascular resistance and thrombosis, which can lead to symptoms. Additionally, people living in cold regions may have increased airway neutrophils, macrophages and airway inflammation [33]. This cold snap can exacerbate inflammation and lead to narrowing of the airways, which can lead to acute asthma attacks [38], but not in children [39] staying indoors of school or home most of the time. Our study showed that cold temperature was related to asthma ERVs compared with MAT and incremental cumulative effects of cold temperature. Our results showed that cold weather was related to respiratory disease in Taoyuan City. Short-term exposure to extremely cold temperatures was associated with asthma ERVs in males and elderly individuals; males worked outside, with more exposure to changes in weather, and elderly individuals had less protection against extreme cold temperature wear less clothes with insensitive to temperature change in outdoor activity.

NO_2_ was mainly from motor vehicle emissions and was not related to asthma ERVs in the four-year period. The potential reason was O_3_ formation through photochemical reactions with NO_2_ and volatile organic compounds at high altitudes [40], which resulted in lower mean daily NO_2_ (12.11 ppm) than that in Taipei (19.83 ppm) [12]. Although we found that NO_2_ was related to asthma ERVs in the warm season, the potential reason was that NO_2_ transferred to O_3_, resulting in higher O_3_ concentrations in the warm season.

We used asthma ERVs of a single regional hospital, urban air pollution and climate data to analyze the association by distributed lag nonlinear time series models. The results demonstrated the harmful effects of short-term exposure to PM_2.5_, ozone and cold temperature on asthma ERVs and discussed the different effects of sex and age.

There were some limitations to our study. First, the daily detailed treatments for asthma were not collected in our study; this may have been related to asthma attacks lasting for days, which may have caused negative effects due to the delay. Second, infection, seasonal change, smoking and in-kitchen smoking were significantly related to acute asthma symptoms and ERVs [26]. Smoking history and second-hand smoke exposure were not checked, which may have increased the number of acute asthma attacks. There was a 13.1% adult smoking population, and approximately 27.1% self-reported second-hand smoke exposure in a smoke survey conducted in 2020 in Taiwan [41]. This needs to be adjusted in future studies. Third, regarding the state of air pollution monitoring by the government, we used the data collected nearest to the hospital rather than to the residence places of the patients who had ERVs, which may overestimate the effect of air pollutants or air temperature. Although Taoyuan Armed Forces General Hospital is a larger hospital in southern Taoyuan that services the nearby residents, the air pollutants near the residence places of the asthma patients need to be checked to increase accuracy in further studies. Fourth, the cumulative effects of PM_2.5_ and O_3_ showed increasing trends, and the cumulative effect over time needs to be evaluated in the future. Fifth, the special terrain of Longtan District and the seasonal winds in Taiwan resulted in increased asthma ERVs, and different areas in Taiwan may have different levels of air pollution. A past study showed that air pollution was related to ERVs of respiratory disease and upper respiratory tract infections in the basin terrain of the Taipei area [42,43]. The relationship between air pollutants and asthma attacks must be studied in other areas with different terrains in the future.

## 5. Conclusions

This study showed positive relationships between short-term air pollutants of fine PM, ozone, climate factors of cold temperature exposure and acute asthma exacerbation. Since the mean fine PM and ozone concentrations have decreased in recent years due to the increased control over the quality of air in Taiwan, adverse effect concentrations have not yet been achieved, according to WHO air pollution guideline values [44], in recent years. The air pollution caused by the development of the semiconductor industry is worth noting. More aggressive policies for environmental pollutant control and asthma attack prevention are needed. This research could play an important role in improving public health policies to accommodate air pollution and low temperatures, which are associated with health risks.

## Figures and Tables

**Table 1:** The asthma emergency room visits, air pollutants and meteorological factors in Longtan district over 4 years (2016–2019).

	Asthma Emergency Room Visits (1321)											
Age	43.43 ± 29.44											
Gender	Males						Females					
	679						642					
Age group (years old)	Children (<18)			Younger (18–44)			Middle-aged (45–64)			Older (>65)		
	423 (32.02%)			228 (17.26%)			272 (20.59%)			378 (30.7%)		
Outcomes	Discharged			Hospitalization			Discharged against medical advice			Mortality		
	999 (75.62%)			296 (22.41%)			19 (1.44%)			1 (0.08%)		
	Median		0.25		0.5		0.75		Minimum		Maximum	
Pm_2.5_ (ppm)	16		11		18.14		23		3		80	
PM_10_ (ppm)	36		27		38.02		47		5		117	
O3 (ppm)	31.5		24.3		32.44		39.02		5.3		77.4	
NO_2_ (ppb)	11.26		8.75		12.11		14.89		1.89		30.46	
CO (ppm)	0.34		0.27		0.3552		0.43		0.08		0.86	
SO_2_ (ppb)	2.4		2		2.438		2.8		0.3		7.4	
Air temperature (°C)	23.03		18.17		22.56		27.61		3.41		32.02	
Relative humidity (%)	76.72		70.72		77.47		84.54		43.93		99.7	

**Table 2:** The correlation between air pollutants and climate factors.

	PM_2.5_	PM_10_	O_3_	NO_2_	SO_2_	CO	AT	RH
PM2.5	1.000	0.884	0.255	0.518	0.461	0.645	−0.105	−0.184
*p*		<0.001	<0.001	<0.001	<0.001	<0.001	<0.001	<0.001
PM_10_	0.884	1	0.24	0.483	0.378	0.575	−0.113	−0.242
*p*	<0.001		<0.001	<0.001	<0.001	<0.001	<0.001	<0.001
O_3_	0.255	0.24	1.000	−0.091	0.096	0.059	−0.25	−0.25
*p*	<0.001	<0.001		<0.001	<0.001	0.025	<0.001	<0.001
NO_2_	0.518	0.483	−0.091	1	0.415	0.846	−0.316	0.195
*p*	<0.001	<0.001	<0.001		<0.001	<0.001	<0.001	<0.001
SO_2_	0.461	0.378	0.096	0.415	1	0.348	0.06	−0.106
*p*	<0.001	<0.001	<0.001	<0.001		<0.001	0.022	<0.001
CO	0.645	0.575	0.059	0.846	0.348	1	−0.432	0.23
*p*	<0.001	<0.001	0.025	<0.001	<0.001		<0.001	<0.001
Air temperature	−0.105	−0.113	−0.25	−0.316	0.06	−0.432	1	−0.39
*p*	<0.001	<0.001	<0.001	<0.001	0.022	<0.001		<0.001
Relative humidity	−0.184	−0.242	−0.25	0.195	−0.106	0.23	−0.39	1
*p*	<0.001	<0.001	<0.001	<0.001	<0.001	<0.001	<0.001	

## Data Availability

The datasets used in the current study are available from the corresponding author upon reasonable request.
